# Unemployment and mental health: a global study of unemployment’s influence on diverse mental disorders

**DOI:** 10.3389/fpubh.2024.1440403

**Published:** 2024-12-13

**Authors:** Yang Yang, Lisi Niu, Saqib Amin, Iftikhar Yasin

**Affiliations:** ^1^Department of Human Resources, Shenzhen Pingle Orthopedic Hospital, Guangdong, Shenzhen, China; ^2^School of Emergency Management, Henan Polytechnic University, Henan, Jiaozuo, China; ^3^Safety and Emergency Management Research Center, Henan Polytechnic University, Henan, Jiaozuo, China; ^4^Department of Economics, Accounting and Finance, University of Oulu, Oulu, Finland; ^5^Department of Government and Public Policy, Faculty of Contemporary Studies, National Defence University, Islamabad, Pakistan

**Keywords:** unemployment, mental health, globalization, institutions, *per capita* income, urbanization, democracy

## Abstract

**Introduction:**

Globally, one in five individuals faces unemployment, which substantially increases their risk of developing mental disorders. Understanding the relationship between unemployment and specific mental health outcomes is crucial for formulating effective policy interventions.

**Methods:**

This study examines the relationship between unemployment and mental disorders across 201 countries from 1970 to 2020. Using a fixed-effects model, we analyze the impact of unemployment on various mental health outcomes, including anxiety, depression, bipolar disorder, drug use, and eating disorders, with a focus on demographic variations.

**Results:**

The analysis reveals a significant positive association between unemployment and mental disorders, particularly anxiety, depression, and bipolar disorder. Moreover, distinct patterns emerge, linking unemployment to higher rates of drug use and eating disorders in specific demographics.

**Discussion:**

These findings underscore the critical interplay between socio-economic factors and mental health, highlighting the need for proactive strategies to address the dual burden of unemployment and mental health disorders. Targeted interventions, such as employment support programs and accessible mental health services, are essential to improve global mental health outcomes. These initiatives can also alleviate the economic burden of unemployment by boosting workforce participation and productivity. Long-term economic gains may offset the increased healthcare expenditures associated with mental health support.

## 1 Introduction

Unemployment is a persistent global challenge with profound socio-economic implications, affecting nearly one in five individuals worldwide. Beyond the loss of income and economic productivity, unemployment is intricately linked to mental health, manifesting in disorders such as anxiety, depression, and bipolar disorder (1, 2). The relationship between unemployment and mental health warrants critical examination, as unemployment not only exacerbates individual mental distress but also poses significant societal and economic burdens. This study explores the multifaceted connection between unemployment and mental disorders, aiming to provide empirical insights that inform targeted policy interventions.

The assertion by the World Health Organization, “no health without mental health,” underscores the intrinsic link between mental wellbeing and overall health (3, 4). Even amidst formidable global health challenges like infectious diseases, the importance of mental health remains resolute (4, 5). As we navigate the pervasive waves of globalization, it becomes increasingly apparent that these societal shifts are intimately entwined with the mental health landscape, particularly concerning unemployment (6). Globalization, underpinned by neoliberal ideologies, redefines economic structures and significantly impacts social frameworks, including the labor market (7). This paradigmatic shift intertwines with mental health, as unemployment emerges as a critical component (8). The economic insecurities and disruptions stemming from unemployment serve as potent stressors, influencing mental health trajectories on a global scale. Furthermore, the globalization of a neoliberal perspective, emphasizing individualism and material pursuits, mirrors the challenges posed by unemployment on mental wellbeing (9).

Mental disorders encompass a complex interplay of abnormal thoughts, perceptions, behaviors, emotions, and relational dynamics (10–12). Within the context of unemployment’s profound impact, studies underscore its adverse effects on mental wellbeing, manifesting as depression, psychosomatic complaints, and diminished self-efficacy (13–17). Unemployment drastically curtails access to critical resources—societal respect, stability, and financial means—intensifying the mental strain experienced by the jobless. Research elucidates that the stigmatization of unemployment significantly impairs mental wellbeing (18–20). Correspondingly, (21) delineate similar psychological effects among the employed populace. Consequently, one’s perception of unemployment emerges as a harbinger of mental health alterations (22).

Urbanization is pivotal in shaping mental health landscapes, as a substantial portion of the global populace resides in urban areas (23). Understanding the ramifications of urbanization on mental wellbeing is crucial, particularly considering the disparities faced by city residents compared to those in rural settings. Studies reveal a 39% increased risk for mood disorders, a 21% increased risk for anxiety disorders, and heightened occurrences of schizophrenia among individuals born and raised in urban environments (24). Moreover, the surge in internal migration has notably exacerbated mental health issues in developed nations, while socio-economic shifts and life events in developing countries have rapidly contributed to the rise in mental disorders (25, 26). Notably, chronic stress, although pivotal for survival, emerges as a predominant risk factor for various neuropsychiatric disorders, encompassing depression, anxiety, and schizophrenia (27).

Profoundly intertwined with future wellbeing, poor childhood mental health perpetuates long-term repercussions, significantly shaping subsequent mental wellbeing (28). Its implications extend beyond mental health, impacting human capital accumulation by diminishing productivity and hindering educational pursuits. Consequently, individuals affected by these mental health challenges encounter enduring struggles across various life domains, notably in employment, income, and other critical outcomes (29, 30).

Mental health issues bear a substantial societal cost, leading to long-term disabilities that burden societies and economies alike (31–33). A staggering 31% of individuals grapple with year-long disabilities stemming from mental health concerns. Disparities in mental health services are evident, with affluent nations typically offering more extensive support compared to low-income countries. Developing nations often face scarcity, with a stark ratio of one psychiatrist per 1 million individuals, limited multidisciplinary teams, and scarce access to essential medications (34). Despite notable efforts and high-profile initiatives, progress in mental health service development remains sluggish, particularly in developing countries (35).

Mental health issues significantly contribute to disability across varying socio-economic landscapes, spanning from developing nations to developed ones (31, 33). These disorders precipitate diverse ailments in developing and developed countries, accentuating the pressing need for a more comprehensive and equitable global approach (36).

Depression casts its shadow over ~264 million individuals worldwide, while psychoses and schizophrenia affect a projected 20 million people (12). However, in developing nations, the mental health of children often remains a neglected concern despite its significant importance. Distinguishing between the mental health challenges faced by adults and children is critical, given their diverse disorder types, risk factors, and required interventions (37). The inadequacy of mental health services in developing countries is a pressing issue, with services often under-resourced, strained, and unable to cater adequately to individuals grappling with mental health issues (38, 39). This recent study explores these nuanced issues, specifically examining their impact on mental health globally.

Unemployment remains a continuous challenge that affects diverse communities despite the attractiveness of economic development. With a global prevalence of about 20%, this condition has far-reaching effects beyond financial losses. It significantly impacts mental wellbeing by triggering an intricate combination of worries, sadness, and numerous mental health illnesses. Hence, this study hypothesizes that unemployment significantly and positively correlates with an increased prevalence of mental disorders, including anxiety, depression, bipolar disorder, drug use, and eating disorders. By testing these hypotheses, this research seeks to illuminate how unemployment impacts mental health and provide evidence for designing effective employment and mental health interventions.

This study makes a novel contribution by offering a comprehensive global analysis of the relationship between unemployment and diverse mental health disorders across 201 countries over five decades. Unlike prior research, which often focuses on specific regions or limited mental health outcomes, this study examines multiple mental disorders, including anxiety, depression, bipolar disorder, drug use, and eating disorders. By integrating demographic variations and employing robust econometric techniques, the study provides deeper insights into the heterogeneity of unemployment’s mental health effects.

The remainder of the study is structured as follows: the subsequent section presents an extensive literature assessment, while the theoretical framework is shown in the third section. Section four offers the technique and the source of data. The fifth portion presents the findings and analysis, while the final section contains the conclusion and policy implications.

## 2 Literature review

Plenty of evidence exists about the association between unemployment and adverse health outcomes (40–42). Taris (22) conducted a study on 229 unemployed Dutch youth and utilized logistic regression, revealing a positive link between unemployment and mental health. However, it found no impact on job search behavior. Backhans and Hemmingsson (43) investigated this relationship further. Their study used logistic regression analysis, covering employed and unemployed individuals from 2001 to 2002. Their findings highlighted that unemployment has a more pronounced impact on men, individuals with high social support or low job control, those working overtime, self-employed individuals, and those with lower occupational class or wages.

Interestingly, among couples without children, unemployment showed the weakest association with mental illness. A study by Pharr et al. (44) finds a significant association between unemployment and mental health issues, but it does not definitively establish causation. A decline in mental health, including depression, anxiety, and stress, is more common among the unemployed. Unemployed people also have a higher risk of developing chronic diseases, including heart disease, hypertension, and musculoskeletal disorders, as well as a higher risk of dying at earlier ages (45). At the same time, additional prospective research has indicated that inadequate mental wellbeing contributes to unemployment (46). Paul and Moser (47) emphasized that the effect of unemployment on mental health depends on the duration of unemployment.

According to Backhans and Hemmingsson (43), people living in couples without kids are less likely to experience psychological difficulties when unemployed. Junna et al. (48) analyzed longitudinal data from Finland from 2008 to 2018, studying 2,720,431 individuals aged 30–60. Their findings revealed that current unemployment significantly impacted worse mental health, especially for men in their 30s with a history of long-term unemployment. This effect was not as pronounced for older men or women. In another study by Wang and Smith (49) covering 418 counties in the Southeastern US in 2017, they discovered a positive relationship between unemployment and mental health indicators across 340 counties. They also found consistent associations between social factors—like lack of post-secondary education, limited health insurance, high housing cost burden, and availability of mental health providers—and various mental health challenges across 345 counties.

Mei et al. (50) addressed the mental health of youth, where experts and adolescent mental health stakeholders convened to establish research priorities. They identified critical gaps in evidence-based research through thematic panel discussions, developing 21 global research priorities on youth mental health. These priorities include public health, innovative interventions, neuroscience, clinical staging, technology, social and cultural determinants, service delivery, translation, and implementation strategies. These priorities aim to pave the way for a systematic and collective plan aimed at improving the global impact of mental health issues among adolescents. Labonté (51) dived into globalization’s intricate impact on international health, particularly post-1980 in the neoliberal era. This exploration scrutinized how globalization affects health through diverse societal channels: healthcare systems, financial reforms, migration, investment treaties, trade, unhealthy goods distribution, labor markets, environmental treaties, and individual rights. The aim is to foster more ethical and sustainable global health outcomes.

Roberts (9) examined how neoliberal globalization impacts global mental health across three key domains: altering material conditions such as employment and inequality, shaping cultural and ideological landscapes affecting individuals’ aspirations and self-identity, and raising challenges in understanding mental illness within diverse global contexts due to the globalization of psychiatry. They underscored the importance of acknowledging and addressing these socio-political determinants as structural factors influencing mental health globally, advocating for open dialogue and proactive engagement among mental health stakeholders to promote universal mental wellbeing while learning from past mistakes.

Moussaoui et al. (52) highlighted the need to moderate the rapid pace of globalization, citing increased ethical responsibilities. They noted positive outcomes like enhanced democracy and adverse effects like mood disorders resulting from this accelerated pace. Ventriglio et al. (53) explored the mental health impact of swift urbanization, linking it to social disparities, pollution, and a disconnection from nature. Chen et al. (54) found that while high urbanization negatively affected physical health at the country level in China between 2000 and 2009, accelerated urbanization positively impacted health due to the healthy migrant phenomenon at sub-provincial levels.

Urbanization’s three dimensions were associated with increased depressive distress, impacting residents’ mental health negatively. Chandra et al. (55) examined urbanization’s effects in India, noting higher substance use and technology addiction among youth, contributing to elevated self-harm rates in various demographic groups like women and elders. Morozov (56) discussed Russia’s perspective, linking modern urbanization to mental health issues arising from stressors like alcohol, smoking, anxiety, and internet addiction, influenced by population pressure, poverty, environmental damage, and social inadequacies. Cornaglia et al. (29) explored the UK’s context, finding a robust negative relationship between educational outcomes and mental health issues through questionnaire data and probit regression analysis.

Wang and Granados (57) studied the impact of China’s economic growth on mental health in individuals aged 45 and above, finding that increasing GDP growth rates were linked to deteriorating mental health indicators, primarily affecting lower-income populations, with similar effects across genders. Heck et al. (58) surveyed mental health services in Alberta’s higher education institutions, revealing a lack of recognition and policies for students with mental health issues. Larger institutions tended to offer more health-related services compared to smaller ones. Hou et al. (59) investigated psychological and physical health’s influence on democracy in Hong Kong, finding that social and personal losses were associated with depressive symptoms, anxiety, poor health, unmarried status, low income, and lower education, suggesting a negative impact of resource depletion on individuals’ wellbeing. Williams (60) studied UK benefit sanctions during the 2010–2015 coalition government, finding a link between Jobseeker’s Allowance (JSA) sanctions and worsened mental health, indicated by increased antidepressant prescriptions. The study urged a reevaluation of sanctions, given their role in the Universal Credit (UC) system and their broader implications for public spending.

Macintyre et al. (61) highlighted the link between poor mental health and socio-economic inequality, arguing that high-income disparity contributes to increased mental illness occurrences. They criticized the predominant focus on psychological and psychiatric perspectives in mental health research and policy, urging a closer examination of socio-economic factors. Their proposal advocated assessing economic policies’ impact on mental health, enhancing professional awareness, fostering community-level partnerships, and supporting socio-economic elements in research and policy. They stressed the need for interdisciplinary approaches to comprehensively address population mental health issues and called for changes in socio-economic policies beyond economic growth to prioritize communal and social welfare considerations.

This recent study aims to bridge gaps in existing research by investigating the relationship between unemployment and mental health globally. It will utilize secondary data to explore how unemployment, globalization, urbanization, GDP growth, children’s education, institutional factors, and democracy collectively impact mental health outcomes. This study stands out by comprehensively analyzing these factors to understand their effects on mental health in various economic contexts, departing from predominantly primary-based studies.

## 3 Theoretical framework

The perception of unemployment significantly influences job search behavior (62–64) highlighted that the extent of active job searching relates positively to one’s intention to find employment. Conversely, satisfaction with the unemployed state negatively impacts job-finding intentions (64). Taris et al. (21) suggest that satisfaction with unemployment is shaped by various current circumstances, such as personal reputation or available finances, ultimately influencing mental health positively. Mental wellbeing can be bolstered by recognition from others, diverse experiences, or financial stability. However, extended periods of joblessness negatively impact mental health (65), leading to feelings of powerlessness that decrease motivation for job searching (22). This diminished motivation reduces the likelihood of successfully finding employment.

According to Albert Bandura’s Social Cognitive Theory, people learn by perusing others and their experiences (66). This theory proposes that in light of unemployment and mental health, people may perceive the negative impacts of job loss on others, leading to increased worry and stress (67). A lack of strong role models in unemployment attempts may reduce self-efficacy, negatively harming mental health. Interventions based on this theory might stress the significance of positive modeling, mentoring, and skill-building programs in increasing people’s confidence in their ability to identify employment (68).

According to the Psychosocial Stress Theory, which was informed by the work of Holmes and Rahe, significant life events such as job loss may cause stress and consequent health problems. As a significant life event, unemployment may create a chain of stress-related reactions that damage mental health (69). Interventions based on this hypothesis may include stress management methods, psychotherapy, and other coping skills to help people deal with the psychological effects of unemployment (70).

## 4 Methodology and data resources

This study adopted a fixed effects methodology to account for individual-specific effects lacking temporal variation that might confound the relationship between unemployment and mental health disorders. While fixed-effects models cannot definitively establish causality (71), they are advantageous in controlling for these unobserved characteristics that could lead to biased estimates (72). Utilizing the fixed-effects approach has several benefits in terms of controlling for unobserved confounding factors that remain constant over time, such as personality traits and genetic predisposition, which are unique to each individual during the study (71). Failure to address these elements may result in erroneous associations between unemployment and mental health. The fixed-effects model effectively differentiates these time-invariant characteristics, allowing us to focus on the within-individual variation in unemployment and its association with changes in mental health.

Initially, a pooled OLS technique was considered but deemed inappropriate due to consistent constants, rejecting the null hypothesis. Subsequently, the Hausman test was subsequently employed to determine the most suitable model between fixed and random effects. The test’s results, with a *p*-value below 5%, indicated the superiority of the fixed effects model for the study, thus guiding its selection for analysis (73).


(1) $$Y_{\mathrm{it}}=\alpha_{i}+\beta_{1}Z_{1\mathrm{it}}+\beta_{2}Z_{2\mathrm{it}}+\beta_{3}Z_{3\mathrm{it}}+\ldots \;\ldots \;\ldots \;\ldots \;\beta_{K}Z_{\mathrm{Kit}}+\in_{\mathrm{it}}$$

To investigate the linkages between unemployment and mental health across the globe, our study employed a standard model Equation 1, drawing from the frameworks proposed by (122, 123).


(2) $$\begin{array}{l} MH_{\mathrm{it}}=\alpha_{i}+\beta_{1}UN_{1\mathrm{it}}+\beta_{2}GLOB_{2\mathrm{it}}+\beta_{3}\mathrm{URBA}N_{3\mathrm{it}}\\ \;+\beta_{4}\mathrm{GDPPC}G_{4\mathrm{it}}+\beta_{5}\mathrm{OSCH}C_{5\mathrm{it}}+\beta_{6}INST_{6\mathrm{it}}\\ \;+\beta_{7}DEMO_{7\mathrm{it}}+\in_{\mathrm{it}} \end{array}$$

The model Equation 2, incorporates various control variables to account for additional factors influencing mental health outcomes. The study incorporated globalization (GLOB) measured using the KOF Globalization Index to account for potential economic, social, and political influences on mental health (74). Urbanization is measured as the percentage of the urban population to control for potential disparities in mental health risks between urban and rural areas (23). Income *per Capita* Growth (GDPPC growth) is measured in constant 2017 international dollars to account for the potential influence of economic wellbeing on mental health (75). Out-of-School Children (OSCHC) are measured as a percentage of the total population to control potential social and educational factors impacting mental health. The Varieties of Democracy (V-Dem) index assesses democracy (DEMO), encompassing multiple dimensions such as electoral, liberal, deliberative, egalitarian, and participatory aspects. To account for potential effects of democratic institutions on mental health outcomes, this variable has been incorporated. Enhanced democratic institutions may exhibit a beneficial impact with improved social safety nets and increased availability of mental health services, hence potentially exerting an influence on the impact of unemployment on mental wellbeing (76).

Data sources include the KOF globalization index, the International Country Risk Guide for institutions, varieties of democracy database v11 for democracy variables, and mental health data from the Institute of Health Metrics and Evaluation (IHME), Global Burden of Disease (GDB), and the WHO. Other variables’ data is sourced from the World Development Indicators, Table 1.

**Table 1:** Variables and data source.

Variables	Description	Data sources
Mental disorders	Mental health disorders	Institute of Health Metrics and Evaluation (IHME), Global Burden of Disease (GDB)
Depressive disorders		
Anxiety disorders		
Alcohol use disorders		
Bipolar disorder		
Drug use disorders		
Eating disorders		
Schizophrenia		
Glob.	Globalization Index	KOF globalization index
GDPPC growth	Growth of *Per Capita* Income	WDI, World Bank
Urban.	Urban population	
Out of school children	Number of children out of school	
Unemployment	Number of unemployed persons	
Inst.	Index of political stability and absence of violence, control of corruption, rule of law, government effectiveness, regulatory quality, and voice and accountability	International Country Risk Guide
Elecdem	Electoral democracy	Varieties of democracy database v11
Liberdem	Liberal democracy	
Delibdem	Deliberative democracy	
Egalitdem	Egalitarian democracy	
Participdem	Participatory democracy	

## 5 Results and discussion

The study relied on well-balanced panel data and considered three primary methods to estimate the model. Pooled regression was initially considered but deemed unsuitable due to unobserved heterogeneity stemming from time and country-specific effects. Consequently, fixed effects and random effects models were preferred. The Hausman test was employed to determine the better fit between random and fixed effects models, revealing a probability value below 5%, confirming the suitability of the fixed effects regression for this model. Furthermore, this study employed the Huber-White estimator to compute robust standard errors, ensuring the reliability of our results under potential violations of classical assumptions, such as heteroskedasticity and serial correlation (77).

Table 2 presents descriptive statistics highlighting the data’s characteristics, central tendencies, and normality, showcasing measures such as mean, median, and mode that signify the data’s central tendency. Tables 3–10 depict the outcomes derived from fixed effects analysis. The R-squared values across these tables indicate a complete explanation of the independent variables by each form of dependent variable.

**Table 2:** Descriptive statistics.

Variable	Mean	Median	Maximum	Minimum	Std.Dev.	Sum	Obs.
Mental disorders	13.38787	13.08905	19.35467	9.467642	1.986063	75895.83	5,669
Depressive disorders	3.968681	3.947907	7.688213	1.640902	0.920955	22264.3	5,610
Anxiety disorders	4.323728	4.070772	9.015948	1.974823	1.22719	24256.11	5,610
Alcohol use disorders	1.587334	1.50806	4.698694	0.3199	0.935285	8904.946	5,610
Bipolar disorder	0.693651	0.601616	1.676204	0.189415	0.249413	3891.381	5,610
Drug use disorders	0.71632	0.616464	3.699504	0.225471	0.424591	4018.556	5,610
Eating disorders	0.212988	0.155819	1.136541	0.045425	0.15796	1194.865	5,610
Schizophrenia	0.278428	0.285798	0.506018	0.191621	0.047067	1561.98	5,610
Unemployment	7.988812	6.68	38.8	0.05	5.755616	33824.63	4,234
Glob.	49.62776	47.15254	90.90649	14.14877	16.7178	444416.6	8,955
GDPPC growth	1.979518	2.112415	140.367	−64.9924	6.011483	16445.83	8,308
Urban.	52.28379	51.8095	100	2.845	24.67649	525033.8	10,042
Out of school children	12.88287	5.3124	89.9454	0	17.47451	57161.29	4,437
Inst	10.18756	0	38.29167	0	12.44197	102374.8	10,049
Elecdem	0.440149	0.383	0.919	0.009	0.289055	3543.198	8,050
Liberdem	0.345441	0.258	0.892	0.005	0.279383	2759.034	7,987
Delibdem	0.344218	0.268	0.887	0.004	0.273248	2770.958	8,050
Egalitdem	0.345611	0.253	0.876	0.015	0.248733	2782.169	8,050
Participdem	0.278645	0.211	0.804	0.006	0.216663	2234.172	8,018

**Table 3:** Fixed effect (FE) estimates of unemployment on mental health.

Variables	Dept. mental disorders							
	(1)	(2)	(3)	(4)	(5)	(6)	(7)	(8)
	Mental disorders	Anxiety disorders	Depress. disorders	Bipolar disorder	Schizo-phrenia	Eating disorders	Drug disorders	Alcohol disorders
Unemployment	0.0094***	0.0057***	0.0137***	−4.59E-	−9.74E***	−0.0003***	−0.0003	−0.0006
	(0.0019)	(0.0010)	(0.0027)	(4.28E-)	(2.07E-)	(0.0001)	(0.0004)	(0.0009)
Constant	13.583	4.5607	3.6743	0.7592	0.2981	0.2628	0.8538	1.8525
	(0.0171)	(0.0091)	(0.0268)	(0.0003)	(0.0001)	(0.0009)	(0.0042)	(0.0085)
Obs.	3,117	3,160	3,160	3,160	3,160	3,160	3,160	3,160
Cross-sections	185	182	182	182	182	182	182	182
*R*^2^	0.981	0.986	0.968	0.999	0.994	0.990	0976	0.974
Time dummy	Yes	Yes	Yes	Yes	Yes	Yes	Yes	Yes
Year dummy	Yes	Yes	Yes	Yes	Yes	Yes	Yes	Yes

***Denotes significance at the 1% level.

**Table 4:** Fixed effect (FE) estimates of unemployment on mental disorders.

Variables	Dept. Mental disorders						
	(1)	(2)	(3)	(4)	(5)	(6)	(7)
Unemployment	0.0132***	0.0132***	0.0137***	0.0136***	0.0135***	0.0135***	0.0137***
	(0.0018)	(0.0018)	(0.0018)	(0.0018)	(0.0018)	(0.0018)	(0.0018)
Glob	−0.0052***	−0.0049***	−0.0052***	−0.0054***	−0.0056***	−0.0054***	−0.0053***
	(0.0018)	(0.0018)	(0.0019)	(0.0019)	(0.0019)	(0.0019)	(0.0019)
GDPPC growth	0.0064***	0.0070***	0.0062***	0.0064***	0.0066***	0.0065***	0.0063***
	(0.0015)	(0.0015)	(0.0016)	(0.0016)	(0.0016)	(0.0016)	(0.0016)
Urban	0.0018	0.0006	0.0027	0.0024	0.0021	0.0021	0.0024
	(0.0023)	(0.0023)	(0.0024)	(0.0024)	(0.0023)	(0.0024)	(0.0024)
Out of school child	0.0020**	0.0018	0.0019	0.0021**	0.0023**	0.0022**	0.0021*
	(0.0011)	(0.0011)	(0.0012)	(0.0012)	(0.0012)	(0.0012)	(0.0012)
Institutions		−0.0055***					
		(0.0011)					
Elecdem			−0.0927				
			(0.0815)				
Liberdem				−0.0018			
				(0.0833)			
Delibdem					0.1117		
					(0.0757)		
Egalitdem						0.1225	
						(0.1145)	
Participdem							−0.0337
							(0.1130)
Constant	13.706***	13.881***	13.738***	13.712***	13.678***	13.671***	13.717***
	(0.1903)	(0.1924)	(0.1973)	(0.1967)	(0.1972)	(0.1996)	(0.1968)
Obs.	1970	1969	1863	1893	1893	1863	1893
Cross-sections	161	161	147	147	147	147	147
*R*^2^	0.991	0.991	0.991	0.991	0.991	0.991	0.991
Time dummy	Yes	Yes	Yes	Yes	Yes	Yes	Yes
Year dummy	Yes	Yes	Yes	Yes	Yes	Yes	Yes

***Denotes significance at the 1% level, **at the 5% level, and *at the 10% level.

**Table 5:** Fixed effect (FE) estimates of unemployment on depressive disorders.

Variables	Dept. depressive disorders						
	(1)	(2)	(3)	(4)	(5)	(6)	(7)
Unemployment	0.0048***	0.0048***	0.0049***	0.0049***	0.0048***	0.0048***	0.0047***
	(0.0012)	(0.0012)	(0.0013)	(0.0013)	(0.0013)	(0.0013)	(0.0013)
Glob	−0.0022**	−0. 0022*	−0.0024*	−0.0024**	−0.0025**	−0.0025**	−0.0025**
	(0.0012)	(0.0012)	(0.0012)	(0.0012)	(0.0012)	(0.0012)	(0.0012)
GDPPC growth	0.0017*	0.0017	0.0018*	0.0019*	0.0020*	0.0020*	0.0019*
	(0.0010)	(0.0010)	(0.0010)	(0.0010)	(0.0010)	(0.0010)	(0.0010)
Urban	0.0101***	0.0101***	0.0105***	0.0104***	0.0104***	0.0102***	0.0103***
	(0.0015)	(0.0015)	(0.0016)	(0.0015)	(0.0015)	(0.0015)	(0.0016)
Out of school child	−0.0008	−0.0008	−0.0008	−0.0007	−0.0007	−0.0006	−0.0007
	(0.0008)	(0.0008)	(0.0008)	(0.0008)	(0.0008)	(0.0008)	(0.0008)
Institutions		0.0001					
		(0.0007)					
Elecdem			0.0169				
			(0.0549)				
Liberdem				0.0539			
				(0.0564)			
Delibdem					0.0967**		
					(0.0512)		
Egalitdem						0.1587**	
						(0.0774)	
Participdem							0.1063
							(0.0759)
Constant	3.2157***	3.2120***	3.1995***	3.1941***	3.1777***	3.1546***	3.1902***
	(0.1283)	(0.1302)	(0.1328)	(0.1324)	(0.1327)	(0.1341)	(0.1324)
Obs.	1972	1971	1895	1895	1895	1895	1895
Cross-sections	160	160	146	146	146	146	146
*R*^2^	0.970	0.970	0.969	0.969	0.969	0.969	0.969
Time dummy	Yes	Yes	Yes	Yes	Yes	Yes	Yes
Year dummy	Yes	Yes	Yes	Yes	Yes	Yes	Yes

***Denotes significance at the 1% level, **at the 5% level, and *at the 10% level.

**Table 6:** Fixed effect (FE) estimates of unemployment on anxiety disorders.

Variables	Dept. anxiety disorders						
	(1)	(2)	(3)	(4)	(5)	(6)	(7)
Unemployment	0.0085***	0.0086***	0.0088***	0.0087***	0.0087***	0.0087***	0.0087***
	(0.0012)	(0.0012)	(0.0013)	(0.0013)	(0.0013)	(0.0013)	(0.0013)
Glob	0.0004	0.0007	0.0004	0.0004	0.0003	0.0004	0.0004
	(0.0012)	(0.0012)	(0.0013)	(0.0012)	(0.0012)	(0.0012)	(0.0013)
GDPPC growth	0.0002	0.0005	0.0001	0.0002	0.0002	0.0003	0.0001
	(0.0010)	(0.0010)	(0.0011)	(0.0011)	(0.0011)	(0.0011)	(0.0010)
Urban	−0.0031**	−0.0037**	−0.0035**	−0.0036**	−0.0036**	−0.0037**	−0.0036**
	(0.0015)	(0.0015)	(0.0016)	(0.0016)	(0.0015)	(0.0016)	(0.0016)
Out of school child	−5.30E-	−0.0001	−7.93E-	−5.01E-	3.00E-	4.49E-	−6.16E-
	(0.0008)	(0.0008)	(0.0008)	(0.0008)	(0.0008)	(0.0008)	(0.0008)
Institutions		−0.0028***					
		(0.0007)					
Elecdem			0.0323				
			(0.0553)				
Liberdem				0.0670			
				(0.0568)			
Delibdem					0.1003*		
					(0.0515)		
Egalitdem						0.1623	
						(0.0779)	
Participdem							0.0865
							(0.0764)
Constant	4.7681***	4.8480***	4.7907***	4.7868***	4.7718***	4.7485***	4.7877***
	(0.1288)	(0.1303)	(0.1337)	(0.1333)	(0.1335)	(0.1350)	(0.1333)
Obs.	1972	1971	1895	1895	1895	1895	1895
Cross-sections	160	160	146	146	146	146	146
*R*^2^	0.990	0.990	0.990	0.990	0.990	0.990	0.990
Time dummy	Yes	Yes	Yes	Yes	Yes	Yes	Yes
Year dummy	Yes	Yes	Yes	Yes	Yes	Yes	Yes

***Denotes significance at the 1% level, **at the 5% level, and *at the 10% level.

**Table 7:** Fixed effect (FE) estimates of unemployment on schizophrenia.

Variables	Dept. schizophrenia						
	(1)	(2)	(3)	(4)	(5)	(6)	(7)
Unemployment	−0.0001***	−0.0001***	−0.0001***	−0.0001***	−0.0001***	−0.0001***	−0.0001***
	(2.80E-)	(2.77E-)	(2.88E-)	(2.88E-)	(2.88E-)	(2.88E-)	(2.89E-)
Glob	−0.0001***	−0.0001***	−0.0001***	−0.0001***	−0.0001***	−0.0001***	−0.0001***
	(2.75E-)	(2.73E-)	(2.84E-)	(2.84E-)	(2.84E-)	(2.83E-)	(2.84E-)
GDPPC growth	−4.01E-*	−5.28E-**	−3.58E-	−3.52E-	−3.37E-	−3.43E-	−3.56E-
	(2.33E-)	(2.31E-)	(2.41E-)	(2.41E-)	(2.41E-)	(2.41E-)	(2.40E-)
Urban	4.50E-	6.79E-**	3.29E-	3.30E-	3.44E-	3.22E-	2.87E-
	(3.38E-)	(3.36E-)	(3.52E-)	(3.51E-)	(3.49E-)	(3.51E-)	(3.52E-)
Out of School Child	6.73E-	8.62E-	6.63E-	5.81E-	7.46E-	6.84E-	6.43E-
	(1.77E-)	(1.75E-)	(1.85E-)	(1.84E-)	(1.84E-)	(1.85E-)	(1.84E-)
Institutions		0.0001***					
		(1.73E-)					
Elecdem			0.0018				
			(0.0012)				
Liberdem				0.0020**			
				(0.0012)			
Delibdem					0.0026**		
					(0.0011)		
Egalitdem						0.0033**	
						(0.0017)	
Participdem							0.0036**
							(0.0016)
Constant	0.2997***	0.2965***	0.2996***	0.2997***	0.2994***	0.2990***	0.2996***
	(0.0028)	(0.0028)	(0.0029)	(0.0029)	(0.0029)	(0.0029)	(0.0029)
Obs.	1972	1971	1895	1895	1895	1895	1895
Cross-sections	160	160	146	146	146	146	146
*R*^2^	0.994	0.994	0.994	0.994	0.994	0.994	0.994
Time dummy	Yes	Yes	Yes	Yes	Yes	Yes	Yes
Year dummy	Yes	Yes	Yes	Yes	Yes	Yes	Yes

***Denotes significance at the 1% level, **at the 5% level, and *at the 10% level.

**Table 8:** Fixed effect (FE) estimates of unemployment on bipolar disorders.

Variables	Dept. bipolar disorders						
	(1)	(2)	(3)	(4)	(5)	(6)	(7)
Unemployment	0.0016**	0.0015**	−0.0001	−0.0002	−5.39E-	−1.48E-	−7.81E-
	(0.0008)	(0.0008)	(0.0007)	(0.0008)	(0.0007)	(0.0008)	(0.0007)
Glob	−0.0002	−0.0001	−0.0001	−0.0002	−0.0002	−0.0001	−0.0001
	(0.0002)	(0.0002)	(0.0002)	(0.0002)	(0.0002)	(0.0002)	(0.0002)
GDPPC growth	−0.0073***	−0.0076***	−0.0059***	−0.0059***	−0.0058***	−0.0062***	−0.0061***
	(0.0011)	(0.0011)	(0.0011)	(0.0011)	(0.0011)	(0.0011)	(0.0011)
Urban	0.0082***	0.0076***	0.0076***	0.0076***	0.0076***	0.0075***	0.0076***
	(0.0002)	(0.0002)	(0.0002)	(0.0002)	(0.0002)	(0.0002)	(0.0002)
Out of school child	0.0012**	0.0010**	0.0030***	0.0029***	0.0028***	0.0032***	0.0030***
	(0.0005)	(0.0005)	(0.0005)	(0.0005)	(0.0005)	(0.0005)	(0.0005)
Institutions		0.0023*					
		(0.0003)					
Elecdem			0.2358***				
			(0.0173)				
Liberdem				0.2138***			
				(0.0167)			
Delibdem					0.2246***		
					(0.0174)		
Egalitdem						0.2326***	
						(0.0188)	
Participdem							0.2852***
							(0.0212)
Constant	0.2547***	0.2363***	0.1387***	0.1832***	0.1748***	0.1749***	0.1712
	(0.0251)	(0.0250)	(0.0249)	(0.0246)	(0.0246)	(0.0247)	(0.0245)
Obs.	1972	1971	1895	1895	1895	1895	1895
Cross-sections	160	160	146	146	146	146	146
*R*^2^	0.947	0.977	0.899	0.865	0.898	0.889	0.898
Time dummy	Yes	Yes	Yes	Yes	Yes	Yes	Yes
Year dummy	Yes	Yes	Yes	Yes	Yes	Yes	Yes

***Denotes significance at the 1% level, **at the 5% level, and *at the 10% level.

**Table 9:** Fixed effect (FE) estimates of unemployment on eating disorders.

Variables	Dept. eating disorders						
	(1)	(2)	(3)	(4)	(5)	(6)	(7)
Unemployment	−0.0002*	−0.0002*	−0.0002*	−0.0002	−0.0002*	−0.0002	−0.0002
	(0.0001)	(0.0001)	(0.0001)	(0.0001)	(0.0001)	(0.0001)	(0.0001)
Glob	−0.0009***	−0.0008***	−0.0008***	−0.0008***	−0.0009***	−0.0009***	−0.0008***
	(0.0001)	(0.0001)	(0.0001)	(0.0001)	(0.0001)	(0.0001)	(0.0001)
GDPPC growth	−0.0007***	−0.0006***	−0.0007***	−0.0007***	−0.0007***	−0.0007***	−0.0007**
	(0.0001)	(0.0001)	(0.0001)	(0.0001)	(0.0001)	(0.0001)	(0.0001)
Urban	−0.0011***	−0.0012***	−0.0012***	−0.0012***	−0.0012***	−0.0012***	−0.0011***
	(0.0001)	(0.0001)	(0.0001)	(0.0001)	(0.0001)	(0.0001)	(0.0001)
Out of school child	0.0003***	0.0003***	0.0002***	0.0002***	0.0002***	0.0002***	0.0002***
	(9.91E-)	(9.88E-)	(0.0001)	(0.0001)	(0.0001)	(0.0001)	(0.0001)
Institutions		−0.0003***					
		(9.75E-)					
Elecdem			−0.0205***				
			(0.0067)				
Liberdem				−0.0216***			
				(0.0068)			
Delibdem					−0.0142**		
					(0.0062)		
Egalitdem						−0.0356***	
						(0.0094)	
Participdem							−0.0389***
							(0.0092)
Constant	0.4024***	0.4116***	0.4175***	0.4162***	0.4162***	0.4234***	0.4173***
	(0.0157)	(0.0159)	(0.0162)	(0.0161)	(0.0162)	(0.0163)	(0.0161)
Obs.	1972	1971	1895	1895	1895	1895	1895
Cross-sections	160	160	146	146	146	146	146
*R*^2^	0.991	0.991	0.991	0.991	0.991	0.991	0.991
Time dummy	Yes	Yes	Yes	Yes	Yes	Yes	Yes
Year dummy	Yes	Yes	Yes	Yes	Yes	Yes	Yes

***Denotes significance at the 1% level, **at the 5% level, and *at the 10% level.

**Table 10:** Fixed effect (FE) estimates of unemployment on drug use disorders.

Variables	Dept. drug use disorders						
	(1)	(2)	(3)	(4)	(5)	(6)	(7)
Unemployment	−0.0027***	−0.0027***	−0.0028***	−0.0028***	−0.0028***	−0.0028***	−0.0028***
	(0.0005)	(0.0005)	(0.0005)	(0.0005)	(0.0005)	(0.0005)	(0.0005)
Glob	−0.0008	−0.0008	−0.0009*	−0.0009*	−0.0009	−0.0009*	−0.0009*
	(0.0005)	(0.0005)	(0.0005)	(0.0005)	(0.0005)	(0.0005)	(0.0005)
GDPPC growth	−0.0010**	−0.0010**	−0.0010**	−0.0010**	−0.0011**	−0.0010**	−0.0010**
	(0.0004)	(0.0004)	(0.0004)	(0.0004)	(0.0004)	(0.0004)	(0.0004)
Urban	−0.0012**	−0.0012**	−0.0013**	−0.0013**	−0.0012*	−0.0013*	−0.0013*
	(0.0006)	(0.0006)	(0.0007)	(0.0007)	(0.0007)	(0.0007)	(0.0007)
Out of school child	0.0001	0.0001	0.0001	0.0001	0.0001	0.0001	0.0001
	(0.0003)	(0.0003)	(0.0003)	(0.0003)	(0.0003)	(0.0003)	(0.0003)
Institutions		4.19E-					
		(0.0003)					
Elecdem			−0.0028				
			(0.0247)				
Liberdem				−0.0087			
				(0.0254)			
Delibdem					−0.0317		
					(0.0231)		
Egalitdem						−0.0052	
						(0.0349)	
Participdem							0.0032
							(0.0342)
Constant	1.0030***	1.0020***	1.0142***	1.0151***	1.0221***	1.0151***	1.0131***
	(0.0578)	(0.0587)	(0.0598)	(0.0596)	(0.0598)	(0.0604)	(0.0596)
Obs.	1972	1971	1895	1895	1895	1895	1895
Cross-sections	160	160	146	146	146	146	146
*R*^2^	0.983	0.983	0.983	0.983	0.983	0.983	0.983
Time dummy	Yes	Yes	Yes	Yes	Yes	Yes	Yes
Year dummy	Yes	Yes	Yes	Yes	Yes	Yes	Yes

***Denotes significance at the 1% level, **at the 5% level, and *at the 10% level.

Table 3 delineates a robust relationship between unemployment and various forms of mental health. Specifically, unemployment demonstrates a positive and significant effect on mental disorders, anxiety disorders, and depressive disorders. Conversely, there is a negative and significant impact of unemployment on bipolar disorder, schizophrenia, eating disorders, drug disorders, and alcohol disorders. The association between the severity of health issues and unemployment could stem from several reasons. Firstly, individuals with severe symptoms may find that their symptoms and the medications used to treat them affect their ability to work (78). Secondly, employers might discern the impact of severity through relapses and performance failures (79). Lastly, individuals with severe health issues might face challenges accessing job support programs (78). Unemployment is a significant source of stress, causing concerns about one’s financial situation, future opportunities, and social status. Research conducted by Lubecka et al. (80) demonstrates a distinct association between unemployment and higher levels of anxiety. Unemployment can significantly contribute to depression by causing a loss of identity, social relationships, and financial security. Research has shown that unemployment is associated with a heightened occurrence of depressive symptoms and major depressive disorder, leading to an increased vulnerability to mental health problems among unemployed individuals (6, 81).

In Tables 4, a robust relationship between unemployment and mental disorders is evident, demonstrating that an increase in global unemployment rates contributes to a rise in mental health issues. Specifically, a 1% increase in unemployment correlates with a 0.0132–0.0137% increase in mental health disorders, a finding consistent with previous studies (26, 78, 82–87). Whitley and Whitley (2) argued that depression, substance abuse, and even suicidal tendencies are linked to unemployment. Unemployed individuals often engage in unhealthy behaviors and lifestyles, increasing the risk of depression (88, 89). Unemployment has been linked to an increased risk of eating disorders and drug use disorders. Research has shown that social adversity, such as economic insecurity, can lead to tobacco use as a response (1). Furthermore, Zimmerman et al. (90) maintained that unemployment is associated with episodes of bipolar disorder.

Regarding globalization, the outcomes demonstrate a detrimental effect on mental health disorders, with a 1% increase in globalization associated with a 0.0049 to 0.0056% decrease in mental health disorders. This aligns with previous research findings (74, 91). The implication is that higher globalization potentially offers more opportunities for growth and prosperity, consequently aiding in reducing mental disorders (40).

Moreover, the study highlights positive associations between GDP *per capita* growth, urbanization, and out-of-school children with mental health disorders. A 1% increase in GDP *per capita* growth, urbanization, and out-of-school children corresponds to a rise of 0.0063 to 0.0070% in mental disorders, respectively. These findings are consistent with prior research on GDP *per capita* growth (40), urbanization (53, 54, 92–94), and out-of-school children (95, 96).

Furthermore, institutions exhibit a negative and significant impact on mental health disorders, while other forms of democracies demonstrate no significant impact.

In Table 5, the relationship between unemployment and depressive disorders is delineated. Unemployment, GDP *per capita* growth, and urbanization exhibit positive and significant effects on depressive disorders, aligning with prior research findings (53, 54, 87, 92, 93). Conversely, globalization and out-of-school children show a negative and significant effect on depressive disorders. A 1% increase in unemployment corresponds to a 0.0047% increase in depressive disorders, consistent with various studies (97–100).

Unemployment contributes to increased mental disorders through various factors, including socio-economic and family-related factors. Economically, reduced income is linked to higher occurrences of mental disorders, particularly among low-income individuals (101–103). Additionally, significant positive relations exist between GDP *per capita* growth and mental health disorders. This might be attributed to the rising income disparities causing reduced self-respect and social harmony, resulting in a depressive effect on mental health (104). Urbanization is linked to increased mental health issues due to polluted and toxic environments (105).

Institutions exhibit a significant positive effect on depressive disorders. Among the democracy variables, only Delibdem and legalities show a positive and substantial connection with depressive disorders, while the others are deemed insignificant.

The findings in Table 6 reveal the relationship between unemployment and anxiety disorders. Unemployment, globalization, and GDP *per capita* growth exhibit a positive and significant impact on anxiety disorders. Conversely, urbanization, out-of-school children, and institutions show a negative effect on anxiety disorders. Precisely, a 1% increase in unemployment corresponds to a 0.0087% increase in anxiety disorders, consistent with various studies (80, 84, 99, 106). Among the democracy variables, all except Delibdem demonstrate insignificant impacts on anxiety disorders, while Delibdem exhibits a positive and significant relation.

The findings in Table 7 showcase the relationship between unemployment and schizophrenia. Unemployment, globalization, and GDP *per capita* exhibit a negative and significant impact on schizophrenia. Specifically, a 1% increase in unemployment decreases schizophrenia issues by 0.0001%, contrary to prior studies (107–111). Conversely, urbanization and out-of-school children contribute to schizophrenia, although not significantly (112, 113). Additionally, institutions and all democracy variables, except Elecdem, significantly correlate with schizophrenia.

The analysis in Table 8 probes the relationship between unemployment and bipolar disorders. Unemployment, urbanization, out-of-school children, institutions, and all democracy variables are significantly associated with bipolar disorders (53, 54, 92, 93, 114). A 1% increase in unemployment leads to a 7.81% rise in bipolar disorders (115–118). Conversely, the remaining variables show a negative and significant impact on bipolar disorders. GDP *per capita* growth displays a negative association, which may suggest that higher economic growth can inadvertently lead to adverse mental health outcomes, particularly in countries with robust GDP growth and robust healthcare systems (40). Additionally, the issue of out-of-school children is linked to health problems, often stemming from factors like poverty, parental unemployment, family violence, and caretaker rejection, which can adversely impact a child’s mental health (114). Maintaining a loving and secure relationship with caregivers during childhood is crucial for children’s mental and physiological wellbeing (119).

In Table 9, the relationship between unemployment and eating disorders is outlined. Most variables demonstrate a significant and detrimental impact on eating disorders, except for out-of-school children. Surprisingly, a 1% increase in unemployment leads to a 0.0002% decrease in eating disorders, a finding that contrasts with prior studies (120, 121). The presence of robust institutions and various democratic forms significantly reduces eating disorders. Well-regulated institutions play a role in mitigating eating disorders by fostering equal opportunities, managing resources effectively, and implementing policies for the public good (124). Additionally, according to (124), good governance diminishes mental health disorders and enhances healthcare systems.

In Table 10, the link between unemployment and drug use disorders is illustrated. Surprisingly, unemployment, globalization, GDP *per capita* growth, and urbanization do not contribute to drug use disorders. However, out-of-school children and institutions show a potential connection, although not significantly. Unexpectedly, a 1% increase in unemployment is associated with a 0.0028% reduction in drug use disorders, which contradicts prior findings (15). Moreover, the variables of democracy, except for one, demonstrate a negative and insignificant impact on drug use disorders.

Table 11 presents the relationship between unemployment and alcohol use disorders. Surprisingly, all variables, except urbanization, display a negative and significant association with alcohol use disorders. Unexpectedly, a 1% increase in unemployment is linked to a 0.0016% reduction in alcohol use disorders, which contradicts previous findings (15, 120, 125).

**Table 11:** Fixed effect (FE) estimates of unemployment on alcohol use disorders.

Variables	Dept. alcohol use disorders						
	(1)	(2)	(3)	(4)	(5)	(6)	(7)
Unemployment	−0.0015	−0.0015	−0.0016	−0.0015	−0.0016	−0.0016	−0.0016
	(0.0011)	(0.0011)	(0.0012)	(0.0012)	(0.0012)	(0.0012)	(0.0012)
Glob	−0.0019*	−0.0020*	−0.0019*	−0.0019	−0.0021*	−0.0021*	−0.0020*
	(0.0011)	(0.0011)	(0.0012)	(0.0012)	(0.0012)	(0.0011)	(0.0012)
GDPPC growth	−0.0002	−0.0004	−0.0003	−0.0003	−0.0001	−0.0001	−0.0002
	(0.0009)	(0.0009)	(0.0010)	(0.0010)	(0.0010)	(0.0010)	(0.0010)
Urban	0.0024*	0.0026*	0.0031**	0.0031**	0.0029**	0.0028**	0.0029**
	(0.0014)	(0.0014)	(0.0014)	(0.0014)	(0.0014)	(0.0014)	(0.0014)
Out of School Child	−0.0008	−0.0008	−0.0008	−0.0008	−0.0007	−0.0007	−0.0008
	(0.0007)	(0.0007)	(0.0007)	(0.0007)	(0.0007)	(0.0007)	(0.0007)
Institutions		0.0011					
		(0.0007)					
Elecdem			−0.0194				
			(0.0511)				
Liberdem				−0.0328			
				(0.0525)			
Delibdem					0.0543		
					(0.0477)		
Egalitdem						0.0925	
						(0.0721)	
Participdem							0.0164
							(0.0706)
Constant	1.8430***	1.8108***	1.8234***	1.8244***	1.8039***	1.7899***	1.8164***
	(0.1192)	(0.1209)	(0.1236)	(0.1233)	(0.1235)	(0.1249)	(0.1232)
Obs.	1972	1971	1895	1895	1895	1895	1895
Cross-sections	160	160	146	146	146	146	146
*R*^2^	0.982	0.982	0.981	0.981	0.981	0.981	0.981
Time dummy	Yes	Yes	Yes	Yes	Yes	Yes	Yes
Year dummy	Yes	Yes	Yes	Yes	Yes	Yes	Yes

***Denotes significance at the 1% level, **at the 5% level, and *at the 10% level.

## 6 Conclusion

Mental health profoundly shapes individuals’ thoughts, actions, and overall wellbeing, which is crucial for a healthy and balanced life free from stress and anxiety. However, numerous factors, including unemployment, significantly impact mental health. The consequences of unemployment, such as financial strain and social pressures, often lead to severe stress and depression. Unfortunately, this situation worsens within a society that lacks robust support systems. Ultimately, unemployment detrimentally affects various aspects of mental health. Globalization and institutional weaknesses contribute negatively, while urbanization and GDP *per capita* exhibit mixed impacts on mental health issues.

The findings call for a multi-pronged approach to address mental health challenges. Policymakers should prioritize integrating mental health considerations into broader socio-economic frameworks, ensuring robust institutional mechanisms and targeted interventions. Future research could explore regional and individual-level variations to offer more nuanced insights, supporting evidence-based policy development tailored to specific contexts.

### 6.1 Limitations

While the fixed-effects methodology accounts for unobserved heterogeneity, it may not fully address endogeneity issues such as reverse causality. Reliance on secondary data may introduce measurement errors, particularly in mental health variables. Additionally, country-level aggregate data may overlook significant regional disparities, limiting generalizability. Finally, data constraints for certain control variables could affect model comprehensiveness. Future research should address these gaps using more granular data and advanced econometric techniques.

## Data Availability

Publicly available datasets were analyzed in this study. This data can be found at: https://www.fao.org/faostat/en/#data/ET, https://ourworldindata.org/mental-health, Global Health Data Exchange | GHDx, https://kof.ethz.ch/en/forecasts-and-indicators/indicators/kof-globalisation-index.html, and https://databank.worldbank.org/. Moreover, authors have not any special rights to access the data.
